# Cardiac characteristics and natural progression in Taiwanese patients with mucopolysaccharidosis III

**DOI:** 10.1186/s13023-019-1112-7

**Published:** 2019-06-13

**Authors:** Hsiang-Yu Lin, Ming-Ren Chen, Shan-Miao Lin, Chung-Lieh Hung, Dau-Ming Niu, Tung-Ming Chang, Chih-Kuang Chuang, Shuan-Pei Lin

**Affiliations:** 1 Department of Medicine, Mackay Medical College, New Taipei City, Taiwan; 2 Department of Pediatrics, Mackay Memorial Hospital, No.92, Sec. 2, Chung-Shan North Road, Taipei, 10449 Taiwan; 3 Department of Medical Research, Mackay Memorial Hospital, 92 Chung-Shan N. Rd., Sec. 2, Taipei, 10449 Taiwan; 4 Mackay Junior College of Medicine, Nursing and Management, Taipei, Taiwan; 5 Department of Medical Research, China Medical University Hospital, China Medical University, Taichung, Taiwan; 6 Institute of Biomedical Sciences, Mackay Medical College, New Taipei City, Taiwan; 7 Division of Cardiology, Department of Internal Medicine, Mackay Memorial Hospital, Taipei, Taiwan; 8 Department of Pediatrics, Taipei Veterans General Hospital, Taipei, Taiwan; 9 Department of Pediatric Neurology, Changhua Christian Children’s Hospital, Changhua, Taiwan; 10 Department of Biological Science and Technology, College of Biological Science and Technology, National Chiao Tung University, Hsinchu, Taiwan; 11 College of Medicine, Fu-Jen Catholic University, Taipei, Taiwan; 12 Department of Infant and Child Care, National Taipei University of Nursing and Health Sciences, Taipei, Taiwan

**Keywords:** Cardiac, Echocardiography, Electrocardiography, Mucopolysaccharidosis III, Valvular heart disease

## Abstract

**Background:**

Mucopolysaccharidosis type III (MPS III), or Sanfilippo syndrome, is caused by a deficiency in one of the four enzymes involved in the lysosomal degradation of heparan sulfate. Cardiac abnormalities have been observed in patients with all types of MPS except MPS IX, however few studies have focused on cardiac alterations in patients with MPS III.

**Methods:**

We reviewed medical records, echocardiograms, and electrocardiograms of 26 Taiwanese patients with MPS III (five with IIIA, 20 with IIIB, and one with IIIC; 14 males and 12 females; median age, 7.4 years; age range, 1.8–26.5 years). The relationships between age and each echocardiographic parameter were analyzed.

**Results:**

Echocardiographic examinations (*n* = 26) revealed that 10 patients (38%) had valvular heart disease. Four (15%) and eight (31%) patients had valvular stenosis or regurgitation, respectively. The most prevalent cardiac valve abnormality was mitral regurgitation (31%), followed by aortic regurgitation (19%). However, most of the cases of valvular heart disease were mild. Three (12%), five (19%) and five (19%) patients had mitral valve prolapse, a thickened interventricular septum, and asymmetric septal hypertrophy, respectively. The severity of aortic regurgitation and the existence of valvular heart disease, aortic valve abnormalities and valvular stenosis were all positively correlated with increasing age (*p* < 0.05). *Z* scores > 2 were identified in 0, 38, 8, and 27% of left ventricular mass index, interventricular septal end-diastolic dimension, left ventricular posterior wall end-diastolic dimension, and aortic diameter, respectively. Electrocardiograms in 11 patients revealed the presence of sinus arrhythmia (*n* = 3), sinus bradycardia (*n* = 2), and sinus tachycardia (*n* = 1). Six patients with MPS IIIB had follow-up echocardiographic data at 1.9–18.1 years to compare with the baseline data, which showed some patients had increased thickness of the interventricular septum, as well as more patients had valvular abnormalities at follow-up.

**Conclusions:**

Cardiac involvement in MPS III is less common and milder compared with other types of MPS. The existence of valvular heart disease, aortic valve abnormalities and valvular stenosis in the patients worsened with increasing age, reinforcing the concept of the progressive nature of this disease.

## Introduction

Mucopolysaccharidoses (MPSs; OMIM 252700) comprise a group of lysosomal storage diseases resulting from deficiencies in specific lysosomal enzymes and involving the sequential degradation of glycosaminoglycans (GAGs), leading to substrate accumulation in various cells and tissues and progressive multi-organ dysfunction. Seven distinct types of MPS disorders (I, II, III, IV, VI, VII, and IX) with 11 specific lysosomal enzyme deficiencies have been reported [1]. The onset and severity of cardiovascular defects are different in each type of MPS, with the most recognized abnormalities being cardiac hypertrophy, cardiac valve thickening, and valvular regurgitation and stenosis [2–13]. Cardiomyopathy and valve defects result from GAG accumulation in the myocardium, cardiac valves, great vessels, and coronary arteries [14]. Deformities in cardiac structures may lead to cardiac dysfunction and mitral or aortic leaflet thickening and calcification resulting in valvular stenosis or regurgitation, which can significantly increase morbidity and mortality [15–18].

MPS III (Sanfilippo syndrome) includes four distinct diseases (types A-D) resulting from a deficiency in one of the four enzymes involved in heparan sulfate degradation as follows: heparan N-sulfatase in type A (OMIM 252900), alpha-N-acetylglucosaminidase (NAGLU) in type B (OMIM 252920), acetyl CoA-alpha-glucosaminide acetyltransferase in type C (OMIM 252930), and N-acetylglucosamine 6-sulfatase in type D (OMIM 252940). MPS III has a variable age of onset and diverse rate of progression characterized by a large phenotypic heterogeneity. Patients with MPS III generally appear unaffected at birth, however clinical manifestations may emerge from 2 to 4 years of age, including intellectual disability, hyperactivity, coarse facial features with broad eyebrows, hirsutism, skeletal dysplasia, degenerative joint disease, hepatosplenomegaly, macrocephaly, and hearing loss [1, 19–21]. Cardiac abnormalities have been observed in patients with all types of MPS, except MPS IX [2–13], however only a few studies have focused on cardiac alterations in patients with MPS III [22–25]. A murine model of MPS IIIB (NAGLU knockout mice, NAGLU^−/−^) demonstrated the development of abnormal valve morphology and function in an age-dependent manner associated with increased myocardial vacuolization, inflammation and fibrosis, as well as a dysregulated lysosomal autophagy in the cardiac tissues [26]. Unlike other MPS diseases, there is neither a satisfactory response to hematopoietic stem cell transplantation nor any available enzyme replacement therapy (ERT) for MPS III. With the development of new disease-modifying treatments such as ERT and gene therapy, it is important to delineate the prevalence and severity of cardiac involvement in this patient population to identify any cardiac complications caused by these experimental therapies. The objective of this study was therefore to investigate the cardiac characteristics and natural progression of MPS III in Taiwanese patients to develop quality of care strategies.

## Materials and methods

### Study population

The medical records, echocardiograms, and electrocardiograms of 26 Taiwanese patients with MPS III (five with IIIA, 20 with IIIB, and one with IIIC; 14 males and 12 females; median age, 7.4 years; age range, 1.8–26.5 years) were retrospectively reviewed at Mackay Memorial Hospital from July 1997 to October 2018. The diagnosis of MPS III was confirmed by measurements of enzymatic activities of particular lysosomal hydrolases in leukocytes or skin fibroblasts, two-dimensional electrophoresis of urinary GAGs, and/or mutational analysis, as well as the exclusion of multiple sulfatase deficiency by the detection of normal enzymatic activities of other lysosomal hydrolases [27, 28]. Six patients with MPS III who had follow-up echocardiographic data at 1.9–18.1 years were also reviewed. The relationships between age and each echocardiographic parameter were analyzed. None of the patients received ERT or a hematopoietic stem cell transplantation during the study period. Written informed consent for cardiac evaluations was obtained from a parent for children and from the patients if they were over 18 years of age. The study was approved by the Ethics Committee of Mackay Memorial Hospital, Taipei, Taiwan.

### Measurements of echocardiographic parameters

We used a Philips Sonos 5500/7500 ultrasound system (Andover, MA, USA) equipped with electronic transducers from 2 to 8 MHz. Data were digitally stored and analyzed by one experienced cardiologist (MRC) to minimize inter-observer variations. Diastolic and systolic diameters were measured using M-mode and two-dimensional echocardiography. The systolic function of the left ventricle was assessed on the basis of the ejection fraction according to the Simpson method. For children, an ejection fraction < 50% was considered abnormal. For adults, an abnormal ejection fraction was defined as < 52% for men and < 54% for women [29]. A shortening fraction < 28% was deemed to be abnormal. Asymmetric septal hypertrophy (ASH) was considered present if left ventricular (LV) interventricular septum/posterior wall thickness ratio in end-diastole ≥1.5 [30]. Diastolic filling was estimated using the E/A ratio by measuring mitral-inflow according to pattern-peak early filling (E) and late filling (A) velocities, and systolic function was assessed using the shortening fraction [31]. A reversed E/A ratio (E/A ratio < 1) was considered to indicate diastolic dysfunction. The severity of valvular stenosis and regurgitation was assessed and graded as follows: 0 (none), 1 (mild), 2 (moderate), and 3 (severe) based on the European Society of Cardiology guidelines [10–12, 32, 33]: mild aortic stenosis (AS) = valve area > 1.5 cm^2^ and mean gradient < 30 mmHg; moderate AS = valve area between 1.0–1.5 cm^2^ and mean gradient between 30 and 50 mmHg; severe AS = valve area < 1.0 cm^2^ and mean gradient > 50 mmHg; mild mitral stenosis (MS) = valve area > 1.5 cm^2^ and mean gradient < 5 mmHg; moderate MS = valve area between 1.0–1.5 cm^2^ and mean gradient between 5 and 10 mmHg; severe MS = valve area < 1.0 cm^2^ and mean gradient > 10 mmHg. As the frequency of physiological tricuspid regurgitation is high in the general population, including the pediatric population, we did not categorize tricuspid regurgitation into pathological findings in this study.

We recorded data on left ventricular mass index (LVMI), right ventricular end-diastolic dimension (RVDd), interventricular septal end-diastolic dimension (IVSd) and end-systolic (IVSs), left ventricular end-diastolic dimension (LVIDd) and end-systolic (LVIDs), left ventricular posterior wall end-diastolic dimension (LVPWd) and end-systolic (LVPWs), aortic diameter, and left atrial dimension (LAD) acquired by echocardiographic evaluations. Relative wall thickness was calculated as (2 × LVPWd)/LVIDd. Concentric remodeling was defined as normal LV mass with relative wall thickness > 0.42 [34]. Measurements of the aorta were made on the sinus from leading edge to leading edge. These values were compared with normal values based on the study of Kampmann et al. [35]. LVMI was computed using the Devereux formula and indexed by height *z* score with normal values according to the report of Foster et al. [36]. All of the aforementioned echocardiographic parameters were transformed into a *z* score derived by subtracting the mean reference value from an individual observed value, and then dividing the difference by the standard deviation from the reference value. A *z* score between − 2 and + 2 was considered to be normal. In addition, 11 patients also had available electrocardiographic (ECG) data. The pediatric values were used as a reference for children.

### Data analysis and statistics

Sex, age, height, weight, and body surface area at the time of echocardiographic assessments were recorded for each patient. Descriptive statistics including means and standard deviations of all echocardiographic parameters were computed. The relationships between age and different echocardiographic parameters were established using Pearson’s correlation coefficient (*r*), and significance was tested using Fisher’s *r–z* transformations. Two-tailed *p*-values were calculated. All statistical analyses were carried out using SPSS version 11.5 (SPSS Inc., Chicago, Illinois, USA). Differences with *p* < 0.05 were considered to be statistically significant.

## Results

**Fig. 1:**
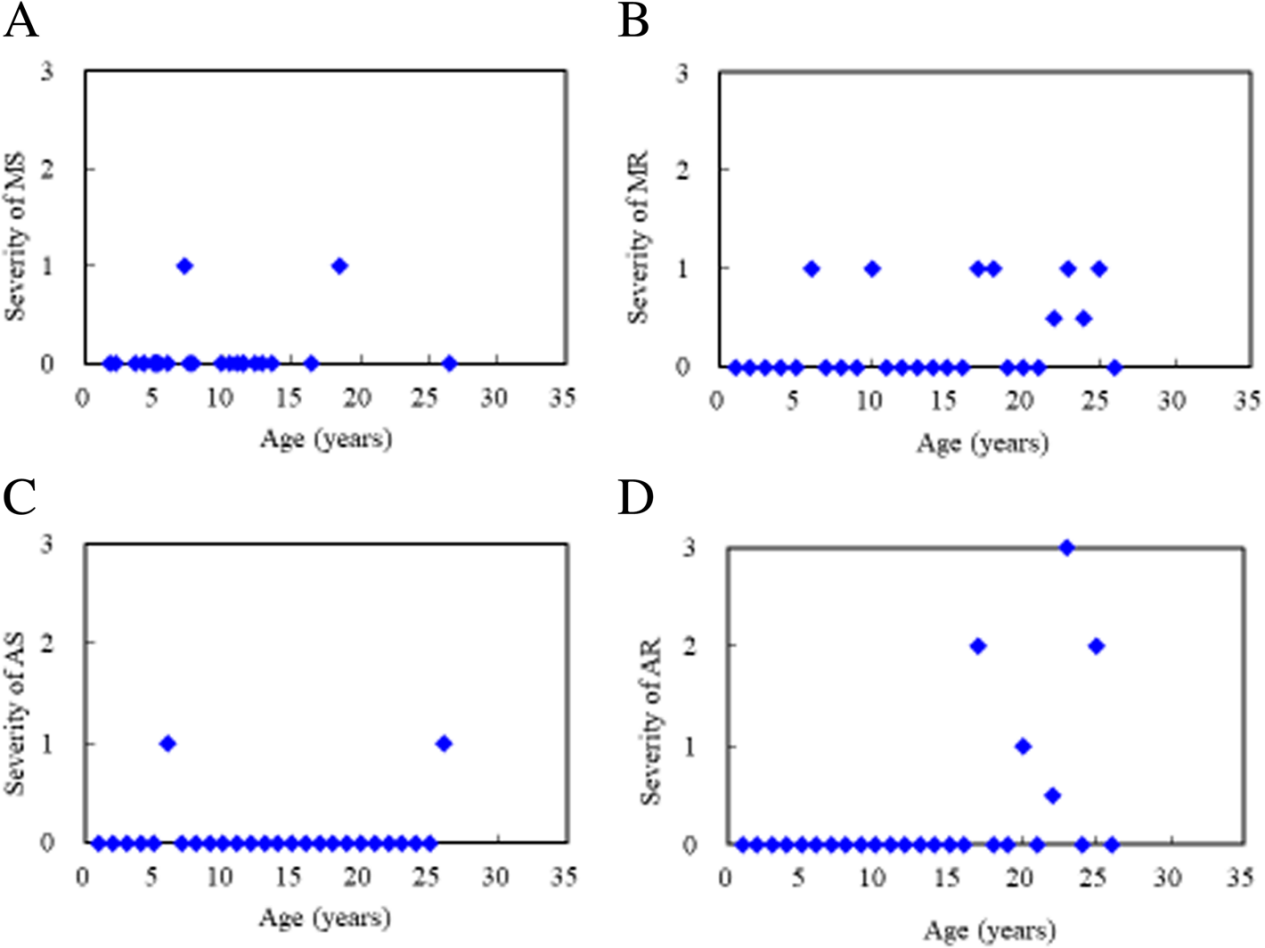
Relationships between age and severity of cardiac valve abnormalities in the 26 patients with MPS III. **a** MS, mitral stenosis; **b** MR, mitral regurgitation; **c** AS, aortic stenosis; **d** AR, aortic regurgitation. Severity of valvular stenosis and regurgitation (MS, MR, AS, AR) were estimated and graded as follows: 0 (none), 1 (mild), 2 (moderate), and 3 (severe)

Tables 1 and 2 show the baseline clinical, echocardiographic and electrocardiographic characteristics of the 26 patients with MPS III. Echocardiographic examinations (*n* = 26) revealed that 10 patients (38%) had valvular heart disease. Four (15%) and eight (31%) patients had valvular stenosis or regurgitation, respectively (Table 3). The most prevalent cardiac valve abnormality was mitral regurgitation (MR) (31%), followed by aortic regurgitation (AR) (19%) (Table 4). However, most cases of valvular heart disease were mild. No one under the age of 4.8 years had valvular stenosis (Fig. 1). Three (12%), five (19%) and five (19%) patients had mitral valve prolapse, a thickened interventricular septum, and ASH, respectively. The severity of aortic regurgitation and the existence of valvular heart disease, aortic valve abnormalities and valvular stenosis were all positively correlated with increasing age (*p* < 0.05) (Tables 3 and 4). The mean *z* scores of LVMI, IVSd, LVPWd, and aortic diameter were − 0.36, 1.71, 0.15 and 1.62, respectively. *Z* scores > 2 were identified in 0, 38, 8, and 27% of LVMI, IVSd, LVPWd, and aortic diameter, respectively (Table 5). Four patients (15%) had left ventricular concentric remodeling (LVMI *z* score < 2 and relative wall thickness > 0.42), and the other 22 patients (85%) had normal LV geometry. E/A ratio < 1 was identified in one patient (4%), however, the ejection fraction and shortening fraction values were normal and revealed no substantial systolic dysfunction. Electrocardiograms in 11 patients revealed the presence of sinus arrhythmia (*n* = 3), sinus bradycardia (*n* = 2), and sinus tachycardia (*n* = 1). The ECG abnormalities were usually of minor clinical significance (Tables 1 and 2). Six patients with MPS IIIB (baseline age range, 1.8 to 5.2 years) had follow-up echocardiographic data at 1.9–18.1 years to compare with the baseline data, and the results showed a change in mean LVMI *z* score from − 0.39 to 0.59, an increase in mean IVSd *z* score from 1.32 to 3.36, a change in mean LVPWd *z* score from 0.13 to 0.24, and changes in mean severity *z* scores of MS, MR, AS, and AR from 0 to 0.5, 0.2 to 0.7, 0 to 0.2, and 0 to 0, respectively (Tables 6 and 7).

**Table 1:** Baseline clinical and echocardiographic features of the 26 patients with MPS III

No.	Gender	MPS type	Age (years)	LVMI (z score)	RVDd (z score)	IVSd (z score)	IVSs (z score)	LVIDd (z score)	LVIDs (z score)	LVPWd (z score)	LVPWs (z score)	AoD (z score)	LAD (z score)	EF (%)	SF (%)	Reversed E/A ratio
1	M	IIIB	1.8	−0.98	**3.20**	**4.08**	1.08	−2.15	− 1.86	− 0.48	− 0.12	0.62	0.14	62%	39%	–
2	M	IIIB	2.2	−0.51	0.67	0.44	0.53	0.70	0.00	−0.43	0.44	1.38	0.14	73%	41%	–
3	F	IIIA	3.6	0.10	**2.73**	**4.07**	0.85	−2.22	−0.85	**2.86**	0.48	**2.53**	1.68	57%	28%	–
4	M	IIIB	4.2	0.44	−1.50	**2.07**	0.41	0.83	−0.04	−0.19	− 0.88	− 0.53	− 1.28	74%	42%	–
5	M	IIIB	4.3	−0.58	NA	−0.11	NA	0.62	0.14	−0.73	NA	1.83	−0.06	69%	38%	–
6	F	IIIB	4.9	−1.73	1.50	**2.29**	−0.17	−1.20	− 0.88	− 0.85	− 0.66	0.65	− 0.88	57%	37%	–
7	M	IIIB	5.1	**−2.31**	−0.05	−1.25	−0.24	0.14	**−2.13**	−0.87	− 0.25	0.71	− 0.55	85%	53%	–
8	M	IIIB	5.1	0.19	−0.60	0.90	1.18	1.97	1.07	−0.57	−0.25	**2.39**	−0.39	54%	38%	–
9	F	IIIB	5.2	0.02	1.19	0.65	0.33	0.13	−1.60	0.02	−0.75	1.35	−0.17	81%	48%	–
10	F	IIIB	5.2	0.28	1.45	1.47	0.54	−0.68	**−2.92**	**2.86**	0.64	1.47	0.36	88%	57%	–
11	M	IIIB	5.4	1.21	0.00	**4.13**	0.35	−0.07	0.64	0.42	1.11	0.67	−0.39	60%	31%	–
12	F	IIIB	6.0	−0.82	1.87	1.31	−1.14	−0.07	0.64	−1.13	−1.39	−0.28	**2.10**	60%	31%	–
13	M	IIIB	7.2	0.36	1.875	**2.63**	1.33	0.41	−0.28	−0.69	**2.59**	0.61	−0.34	72%	41%	–
14	M	IIIB	7.5	0.58	1.65	**3.24**	0.78	−0.89	−0.79	1.38	−0.93	0.72	0.90	59%	38%	–
15	M	IIIB	7.8	1.10	1.29	2.00	−0.48	1.26	1.38	0.52	−0.79	**2.83**	−1.03	54%	34%	–
16	M	IIIA	9.9	−1.54	−1.28	− 1.04	−0.15	1.44	0.04	−1.51	−0.76	1.83	1.26	74%	43%	–
17	F	IIIB	10.5	−0.40	NA	1.37	NA	−0.03	−0.61	0.75	NA	1.88	−0.60	72%	40%	–
18	F	IIIA	11.0	−1.61	0.96	**2.44**	0.90	−1.51	−0.68	−0.44	− 1.26	0.56	− 1.35	63%	33%	–
19	F	IIIB	11.4	0.50	NA	**5.56**	0.83	−1.52	−0.57	0.31	−0.13	**2.86**	−1.57	60%	31%	–
20	F	IIIB	11.5	−0.09	−0.09	0.11	0.04	0.55	−0.89	0.43	1.27	0.06	0.63	77%	45%	–
21	F	IIIB	12.3	−1.48	**2.04**	**2.03**	0.56	−0.12	−0.38	−1.13	−0.44	**3.56**	−0.72	70%	39%	–
22	F	IIIB	12.9	−1.60	NA	0.11	NA	−1.74	**−2.32**	0.38	NA	1.26	**−2.75**	75%	43%	–
23	M	IIIA	13.6	0.50	NA	0.91	NA	0.97	−0.20	0.36	NA	1.74	−1.52	73%	42%	–
24	M	IIIA	16.4	−0.20	NA	2.00	NA	0.00	−0.25	0.92	NA	**5.19**	**−4.29**	68%	38%	–
25	F	IIIB	18.5	0.23	NA	1.33	NA	1.62	1.24	1.73	NA	**6.17**	−0.34	66%	36%	**+**
26	M	IIIC	26.5	−1.13	−1.76	1.74	0.23	0.00	0.54	0.04	−1.71	0.12	−0.44	63%	33%	–

*MPS* Mucopolysaccharidosis, *LVMI* Left ventricular mass index, *RVDd* Right ventricular end-diastolic dimension, *IVSd* Interventricular septal end-diastolic dimension, *IVSs* Interventricular septal end-systolic dimension, *LVIDd* Left ventricular end-diastolic dimension, *LVIDs* Left ventricular end-systolic dimension, *LVPWd* Left ventricular posterior wall end-diastolic dimension, *LVPWs* Left ventricular posterior wall end-systolic dimension, *AoD* Aortic diameter, *LAD* Left atrial dimension, *EF* Ejection fraction, *SF* Shortening fraction, *E/A* Ratio between early and late (atrial) ventricular filling velocity, *NA* Not available

The abnormal values (z score >2 or <-2) are presented in boldface

**Table 2:** Baseline clinical, echocardiographic and electrocardiographic features of the 26 patients with MPS III

No.	Gender	MPS type	Age (years)	MS	MR	AS	AR	MVP	Thick IVS	ASH	Left ventricular remodeling pattern	Electrocardiographic features
1	M	IIIB	1.8	0	0	0	0	–	+	+	Concentric remodeling	Normal
2	M	IIIB	2.2	0	0	0	0	–	–	–	Normal	Normal
3	F	IIIA	3.6	0	0	0	0	–	+	–	Concentric remodeling	NA
4	M	IIIB	4.2	0	0	0	0	–	–	–	Normal	Normal
5	M	IIIB	4.3	0	0	0	0	–	–	–	Normal	Sinus bradycardia, sinus arrhythmia
6	F	IIIB	4.9	0	1	1	0	–	–	+	Normal	Normal sinus rhythm, borderline QTc
7	M	IIIB	5.1	0	0	0	0	–	–	–	Normal	NA
8	M	IIIB	5.1	0	0	0	0	–	–	–	Normal	NA
9	F	IIIB	5.2	0	0	0	0	–	–	–	Normal	Sinus arrhythmia
10	F	IIIB	5.2	0	1	0	0	+	–	–	Normal	Sinus tachycardia
11	M	IIIB	5.4	0	0	0	0	–	–	–	Normal	NA
12	F	IIIB	6.0	0	0	0	0	+	–	–	Normal	NA
13	M	IIIB	7.2	1	0	0	0	–	+	+	Normal	NA
14	M	IIIB	7.5	0	0	0	0	+	–	–	Concentric remodeling	NA
15	M	IIIB	7.8	0	0	0	0	–	+	–	Normal	NA
16	M	IIIA	9.9	0	0	0	0	–	–	–	Normal	Sinus arrhythmia
17	F	IIIB	10.5	0	1	0	2	–	–	–	Normal	NA
18	F	IIIA	11.0	0	1	0	0	–	–	–	Normal	NA
19	F	IIIB	11.4	0	0	0	0	–	+	+	Concentric remodeling	NA
20	F	IIIB	11.5	0	0	0	1	–	–	–	Normal	NA
21	F	IIIB	12.3	0	0	0	0	–	–	+	Normal	Sinus bradycardia, short PR interval
22	F	IIIB	12.9	0	0.5	0	0.5	–	–	–	Normal	NA
23	M	IIIA	13.6	0	1	0	3	–	–	–	Normal	NA
24	M	IIIA	16.4	0	0.5	0	0	–	–	–	Normal	NA
25	F	IIIB	18.5	1	1	0	2	–	–	–	Normal	Normal
26	M	IIIC	26.5	0	0	1	0	–	–	–	Normal	Normal

*MPS* Mucopolysaccharidosis, *MS* Mitral stenosis, *MR* Mitral regurgitation, *AS* Aortic stenosis, *AR* Aortic regurgitation, *MVP* Mitral valve prolapse, *IVS* Interventricular septum, *ASH* Asymmetric septal hypertrophy, *NA* Not available. Severity of valvular stenosis and regurgitation (MS, MR, AS, AR) were estimated and graded on the following scores: 0 (none), 1 (mild), 2 (moderate), and 3 (severe)

**Table 3:** Echocardiographic features of the 26 patients with MPS III and the relationships between cardiac valve abnormalities and age

Cardiac valve abnormalities	Valvular heart disease	Valvular stenosis	Valvular regurgitation	Mitral valve abnormality	Aortic valve abnormality
n (%)	10 (38%)	4 (15%)	8 (31%)	9 (35%)	7 (27%)
*r* value (cardiac valve abnormalities versus age)	0.554	0.419	0.343	0.302	0.572
*p* value	***p <*** **0.01**	***p <*** **0.05**	*p* > 0.05	*p* > 0.05	***p <*** **0.01**

*MPS* Mucopolysaccharidosis

*p* value <0.05 and *p* value <0.01 are presented in boldface

**Table 4:** Echocardiographic features of the 26 patients with MPS III and the relationships between severity of cardiac valve abnormalities and age

Echocardiographic features	MS	MR	AS	AR	MVP	Thick IVS
n (%)	2 (8%)	8 (31%)	2 (8%)	5 (19%)	3 (12%)	5 (19%)
*r* value (severity of cardiac valve abnormalities versus age)	0.211	0.272	0.357	0.375	−0.171	−0.218
*p* value	*p* > 0.05	*p* > 0.05	*p >* 0.05	***p*** **< 0.05**	*p* > 0.05	*p* > 0.05

*MPS* Mucopolysaccharidosis, *MS* Mitral stenosis, *MR* Mitral regurgitation, *AS* Aortic stenosis, *AR* Aortic regurgitation, *MVP* Mitral valve prolapse, *IVS* Interventricular septum

*p* value <0.05 is presented in boldface

**Table 5:** The values of echocardiographic parameters of the 26 patients with MPS III

Echocardiographic parameters	LVMI (*z* score)	RVDd (*z* score)	IVSd (*z* score)	IVSs (*z* score)	LVIDd (*z* score)	LVIDs (*z* score)	LVPWd (*z* score)	LVPWs (*z* score)	AoD (*z* score)	LAD (*z* score)
Mean	−0.36	0.80	1.71	0.39	−0.06	−0.44	0.15	−0.19	1.62	−0.44
SD	0.94	1.41	1.62	0.61	1.15	1.09	1.12	1.03	1.57	1.31
Z score > 2 (%)	0%	16%	38%	0%	0%	0%	8%	5%	27%	4%

*MPS* Mucopolysaccharidosis, *LVMI* Left ventricular mass index, *RVDd* Right ventricular end-diastolic dimension, *IVSd* Interventricular septal end-diastolic dimension, *IVSs* Interventricular septal end-systolic dimension, *LVIDd* Left ventricular end-diastolic dimension, *LVIDs* Left ventricular end-systolic dimension, *LVPWd* Left ventricular posterior wall end-diastolic dimension, *LVPWs* Left ventricular posterior wall end-systolic dimension, *AoD* Aortic diameter, *LAD* Left atrial dimension, *SD* Standard deviation

**Table 6:** Six patients with MPS IIIB who had follow-up echocardiographic examinations after 1.9–18.1 years of follow-up compared with the baseline data

MPS type	Gender	Age at baseline (years)	Age at follow-up (years)	Duration (years)	LVMI (z score)		Change (z score)	IVSd (z score)		Change (z score)	LVPWd (z score)		Change (z score)
					Baseline	Follow-up		Baseline	Follow-up		Baseline	Follow-up	
IIIB	F	5.2	23.3	18.1	0.02	0.99	0.97	0.65	8.11	7.46	0.02	−0.19	−0.21
IIIB	F	5.2	23.3	18.1	0.28	1.06	0.78	1.47	3.32	1.86	2.86	0.80	−2.06
IIIB	M	5.1	7.8	2.7	−2.31	−0.16	2.15	−1.25	1.18	2.43	−0.87	0.53	1.39
IIIB	M	5.1	9.3	4.2	0.19	0.27	0.08	0.90	1.40	0.50	−0.57	0.51	1.08
IIIB	M	4.2	6.8	2.6	0.44	0.24	−0.20	2.07	1.38	−0.69	−0.19	− 0.63	−0.44
IIIB	M	1.8	3.7	1.9	−0.98	1.11	2.09	4.08	4.76	0.68	−0.48	0.41	0.89
Mean		4.4	12.4	8.0	−0.39	0.59	0.98	1.32	3.36	2.04	0.13	0.24	0.11

*MPS* Mucopolysaccharidosis, *LVMI* Left ventricular mass index, *IVSd* Interventricular septal end-diastolic dimension, *LVPWd* Left ventricular posterior wall end-diastolic dimension

**Table 7:** Six patients with MPS IIIB who had follow-up echocardiographic examinations after 1.9–18.1 years of follow-up compared with the baseline data

MPS type	Gender	Age at baseline (years)	Age at follow-up (years)	Duration (years)	Severity score of MS		Change of severity score	Severity score of MR		Change of severity score	Severity score of AS		Change of severity score	Severity score of AR		Change of severity score
					Baseline	Follow-up		Baseline	Follow-up		Baseline	Follow-up		Baseline	Follow-up	
IIIB	F	5.2	23.3	18.1	0	1	1	0	1.5	2	0	0	0	0	0	0
IIIB	F	5.2	23.3	18.1	0	1	1	1	1.5	0.5	0	0	0	0	0	0
IIIB	M	5.1	7.8	2.7	0	0	0	0	0	0	0	0	0	0	0	0
IIIB	M	5.1	9.3	4.2	0	1	1	0	0	0	0	1	1	0	0	0
IIIB	M	4.2	6.8	2.6	0	0	0	0	1	1	0	0	0	0	0	0
IIIB	M	1.8	3.7	1.9	0	0	0	0	0	0	0	0	0	0	0	0
Mean		4.4	12.4	8.0	0	0.5	0.5	0.2	0.7	0.5	0	0.2	0.2	0	0	0

*MPS* Mucopolysaccharidosis, *MS* Mitral stenosis, *MR* Mitral regurgitation, *AS* Aortic stenosis, *AR* Aortic regurgitation. Severity of valvular stenosis and regurgitation (MS, MR, AS, AR) were estimated and graded on the following scores: 0 (none), 1 (mild), 2 (moderate), and 3 (severe)

## Discussion

As far as we are aware, this is the first study to delineate the cardiac structure and function and natural progression of MPS III in Asian patients and compare them with normal values obtained from a population that included young adults on the basis of the report of Kampmann et al. [35]. Compared with the other types of MPS diseases, cardiac involvement in MPS III has received relatively little attention [22–25]. Our results demonstrated that most of the patients with MPS III had mild valvular heart disease, and some had aortic dilatation and increased thickness of the interventricular septum. We found an increase in IVSd, however, no increase in LVMI. IVSd is part of the LVMI. The clinical relevance of an isolated increased IVSd might be due to LV remodeling pattern of these patients. In this cohort, four patients (15%) had LV concentric remodeling defined as normal LV mass with relative wall thickness > 0.42 [34]. The valvular stenosis in these patients worsened with increasing age, in accordance with the progressive nature of this disease. For the six patients with MPS IIIB who had follow-up echocardiographic data at 1.9–18.1 years, echocardiography showed some patients had increased thickness of the interventricular septum, as well as more patients had valvular abnormalities at follow-up. Our results are consistent with those of a previous study in a Caucasian population [22].

Cardiac involvement in MPS III has been reported to be less common and milder compared with the other types of MPS [2, 10, 20–23, 37]. Nijmeijer et al. [23] reported mitral valve abnormalities and aortic valve abnormalities in 13/30 (43%) and 10/30 (33%) patients with MPS III, respectively. Consistently, echocardiographic examinations in our cohort also revealed that 35 and 27% of the patients had mitral valve abnormalities or aortic valve abnormalities, respectively. In this study, 38% of the MPS III patients had valvular heart disease, however most of the cases had mild disease, and no one under the age of 4.8 years had valvular stenosis.

Echocardiographic assessments revealed mean *z* scores of LVMI, IVSd, LVPWd, and aortic diameter of − 0.36, 1.71, 0.15 and 1.62, respectively, and z scores > 2 were identified in 0, 38, 8, and 27% of LVMI, IVSd, LVPWd, and aortic diameter, respectively. Bolourchi et al. [38] reported that patients with MPS III had a high prevalence of aortic root dilatation (3/6, 50%), which is consistent with our results (7/26, 27%). Although LV systolic function according to ejection fraction and shortening fraction values was normal in all of our patients, however, ejection fraction and shortening fraction values are parameters that show abnormalities when there is substantial LV dysfunction. Speckle-tracking echocardiography is a marker for early, subclinical LV dysfunction which was recently reported in patients with MPS III by Nijmeijer et al. [23]. In our study, a reversed E/A ratio (< 1) was identified in one patient (4%). However, an abnormal mitral valve E/A ratio could also be attributed to mitral valve abnormalities. This patient (No. 25) with the abnormal E/A ratio also had mitral valve abnormalities. Thus this could not definitely be attributed to diastolic dysfunction. Previous studies have indicated that abnormal catabolism of dermatan sulfate in patients with MPS I, II and VI results in the accumulation of dermatan-sulfated GAGs in cardiac valves, leading to valvular thickening and other cardiac defects [6, 7]. The main storage products of MPS III is heparan sulfate, which has been reported to potentially be an essential constituent of life-long cardiac conduction system plasticity and that its storage results in atrioventricular block [39]. Cardiac lesions may be less prominent in MPS III than in MPS I, II, and VI [37]. Aortic dilatation and increased interventricular septum thickness, as well as valvular stenosis and regurgitation were still present in some of our patients, and the severity of aortic regurgitation also worsened with increasing age.

There were varying degrees of valvular deformities in our patients, although most had mild stenosis or regurgitation. Valvular regurgitation (31%) was more common than valvular stenosis (15%) in our cohort, which is consistent with the study of Wilhelm et al. [22]. They also reported that left-sided valves were much more commonly involved than right-sided valves in patients with MPS III. In our study, the most prevalent cardiac valve abnormality was MR (31%), followed by AR (19%). In relation to grade 1 MR, Kampmann et al. [40] described that it was a common finding in the pediatric population in their experience, thus they did not consider MR grade 1 in their study results of valve abnormalities in MPS II. However, in the few original studies focusing on cardiac alterations in patients with MPS III by Wilhelm et al. [22] and Nijmeijer et al. [23], they both reported the findings of grade 1 MR in their results. Our report was consistent with the latter studies.

Ventricular remodeling indicates alterations in ventricular architecture with associated increases in volume and altered chamber configuration leading to myocyte hypertrophy and apoptosis, myofibroblast proliferation, and interstitial fibrosis [41]. Few reports have described the LV remodeling pattern in patients with MPS. In our study, four patients (15%) had LV concentric remodeling associated with a higher risk of subsequent cardiovascular events compared to the other 22 patients (85%) with normal LV geometry.

Forty-five percent of our ECGs showed specific findings, including sinus arrhythmia (3/11, 27%), sinus bradycardia (*n* = 2), and sinus tachycardia (*n* = 1) although the clinical significance was minor. However, a respiratory arrhythmia is common amongst children. Thus these findings did not have to be pathological. Sudden and unexpected death due to heart block has been reported in isolated case reports of adults with MPS II, III and VI [13, 39]. Although ECG has been reported to be an unreliable tool for detecting cardiologic defects in MPS [9], due to the rapidity and easy accessibility of this inexpensive diagnostic tool, we still suggest that ECG should remain part of the follow-up examinations of patients with MPS III, especially to identify rhythm abnormalities or changes in conduction.

ERT for other MPS diseases appears to be effective in stabilizing or reducing cardiac hypertrophy, and better results may be associated with starting ERT at a younger age. There is no sufficient evidence to state an effect of ERT on valvulopathy. Some reports might show that ERT appears to diminish deterioration of already developed valvular heart disease [11, 12, 42], however, some studies report deterioration of valvulopathy or an increase in number of patients with valvulopathy after ERT [9, 14]. Further studies are needed to elucidate whether successful gene therapy can lead to similar cardiac outcomes. Due to the progressive nature of MPS, initiating ERT or gene therapy before the occurrence of irreversible cardiac damage may contribute to a better clinical outcome. Thus, making an early diagnosis through screening programs for high-risk populations or newborns is very important [43–46].

### Limitations

As a retrospective and uncontrolled study, there was no healthy control group to compare the echocardiographic parameters with those of our patients. Not all of the patients in this cohort had follow-up echocardiographic data to compare with baseline data. We used the reference values from the Caucasian population due to the lack of those from the Asian population. Although the patients in this cohort were included from 1997 and onwards, all the images from echocardiographs from 1997 had sufficient quality with reliable and reproducible measurement. The small sample size of patients with MPS III reflects the rare nature of this genetic disorder. In addition, both the degree of disease severity and age range (1.8–26.5 years) varied considerably. As a result, studies with larger cohorts and longer follow-up periods are warranted.

## Conclusion

Cardiac involvement in MPS III is less common and milder compared with the other types of MPS. In this study, a substantial proportion of the patients with MPS III had aortic dilatation, increased interventricular septum thickness, and mild valvular heart disease. Our six MPS IIIB patients had worse valvular heart disease and cardiac hypertrophy according to echocardiographic examinations performed after 1.9–18.1 years of follow-up. The aortic valve abnormalities and valvular stenosis in these patients worsened with increasing age, which is consistent with the progressive nature of this disease. Thus, it is crucial to make an early diagnosis through screening programs for high-risk populations or newborns in order to initiate ERT or gene therapy before the occurrence of irreversible cardiac damage to improve the clinical outcome. These findings and follow-up data can also be used to develop quality of care strategies for such patients.

## Data Availability

Not applicable. There are no other supporting data and materials since all of them are in this article.

## Abbreviations

AR: Aortic regurgitation
AS: Aortic stenosis
ASH: Asymmetric septal hypertrophy
E/A: Ratio between early and late (atrial) ventricular filling velocity
ECG: Electrocardiography
ERT: Enzyme replacement therapy
GAGs: Glycosaminoglycans
IVSd: Interventricular septal end-diastolic dimension
IVSs: Interventricular septal end-systolic dimension
LAD: Left atrial dimension
LV: Left ventricular
LVIDd: Left ventricular end-diastolic dimension
LVIDs: Left ventricular end-systolic dimension
LVMI: Left ventricular mass index
LVPWd: Left ventricular posterior wall end-diastolic dimension
LVPWs: Left ventricular posterior wall end-systolic dimension
MPS: Mucopolysaccharidosis
MR: Mitral regurgitation
MS: Mitral stenosis
NAGLU: Alpha-N-acetylglucosaminidase
RVDd: Right ventricular end-diastolic dimension
